# Serrodyne‐Enabled Dual Electro‐Optic Comb Interferometry for High‐Precision Absolute Ranging and Integration‐Ready Metrology

**DOI:** 10.1002/advs.202507459

**Published:** 2025-07-30

**Authors:** Xiaoyang Guo, Xusheng Yang, Jiawen Zhi, Chenggang Shao, Hanzhong Wu

**Affiliations:** ^1^ National Gravitation Laboratory MOE Key Laboratory of Fundamental Physical Quantities Measurement School of Physics Huazhong University of Science and Technology Wuhan 430074 China

**Keywords:** dual electro‐optic frequency combs, high‐precision absolute distance measurement, nanometric vibration sensing, photonic integration, serrodyne modulation

## Abstract

Optical frequency combs (OFCs) have revolutionized precision metrology, enabling highly precise frequency and distance measurements. Dual electro‐optic frequency comb ranging systems traditionally rely on acousto‐optic modulators (AOMs) to shift the local oscillator frequency, mitigating frequency degeneracy but restricting tuning flexibility, response speed, and power efficiency. Here, an AOM‐free dual electro‐optic frequency comb ranging system is introduced, employing a serrodyne‐modulated electro‐optic modulator (EOM) for frequency shifting, achieving superior phase coherence and high‐precision distance measurement. Experimental validation confirms nanometric ranging precision, with Allan deviation below 0.1 nm at 1 ms integration. The system effectively tracks high‐frequency vibrations (up to 100 kHz) from a piezoelectric transducer and enables dynamic 3D surface imaging. Moreover, it detects nanoscale water surface vibrations through precise laser‐ranging analysis. Notably, the system maintains high measurement precision across a wide spatial scale—from meter‐level free‐space ranging to nanometer‐scale vibration sensing—demonstrating exceptional versatility. Compared to conventional AOM‐based approaches, the method provides enhanced flexibility, reduced RF power consumption, and improved photonic integration compatibility, thus offering substantial benefits for precision metrology and high‐resolution sensing applications.

## Graphical abstract

A serrodyne‐modulated dual electro‐optic comb interferometer is presented that achieves sub‐nanometer precision in absolute distance measurements. The system also supports diverse applications such as depth imaging and vibration sensing. With features of low RF power, wide frequency‐shifting range, and full integration potential, the system offers a feasible path toward compact, fast, and power‐efficient laser ranging for next‐generation photonic sensing applications.

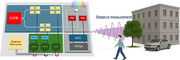

## 1 Introduction

The optical frequency comb (OFC) enables unprecedented precision in time and frequency measurements, facilitating high‐resolution spectroscopy^[^ ^1^, ^2^, ^3^, ^4^, ^5^, ^6^ ^]^ and metrology.^[^ ^7^, ^8^, ^9^, ^10^, ^11^, ^12^, ^13^, ^14^, ^15^, ^16^, ^17^, ^18^ ^]^ The key advantages of optical frequency combs over conventional methods lie in their exceptional precision and rapid measurement capabilities, making them invaluable tools for a wide range of scientific and engineering applications. With the advancement of integrated photonics, the integration of waveguide‐based electro‐optic modulators (EOMs) into silicon photonic circuits has become feasible.^[^ ^19^, ^20^, ^21^, ^22^, ^23^ ^]^ Photonic integrated circuit (PIC) technology holds immense potential for enabling low‐cost, high‐precision on‐site measurement. Electro‐optic frequency combs based on on‐chip modulators combine integration capabilities with great frequency agility and straightforward generation.^[^ ^24^, ^25^, ^26^, ^27^, ^28^, ^29^, ^30^, ^31^, ^32^, ^33^ ^]^ This makes them well‐suited for high‐resolution measurements in a variety of precision sensing applications. However, in dual electro‐optic frequency comb interferometry systems, while two electro‐optic combs with distinct repetition and offset frequencies can be employed, a shared seed laser is often utilized to generate two combs with synchronized coherence. This approach, particularly common in dual electro‐optic comb configurations, necessitates an acousto‐optic modulator (AOM) at the local oscillator (LO) to spectrally shift the beat frequencies of the positive and negative sidebands away from DC,^[^ ^34^, ^35^, ^36^, ^37^ ^]^ thereby resolving frequency degeneracy in multi‐heterodyne signals. While integrated acousto‐optic modulators based on thin‐film lithium niobate (TFLN) have been demonstrated as viable,^[^ ^38^ ^]^ platforms using purely electro‐optic methods offer lower manufacturing complexity. Therefore, using EOMs to achieve frequency shifting at the LO presents a preferable solution.

In contrast to acousto‐optic modulators, which rely on the acousto‐optic effect for frequency shifting, serrodyne modulation achieved through EOMs can also effectively shift the carrier frequency.^[^ ^39^ ^]^ This is accomplished by applying a ramp‐phase modulation in the time domain, a technique widely employed for frequency translation in RF,^[^ ^40^, ^41^ ^]^ microwave,^[^ ^40^, ^42^, ^43^, ^44^ ^]^ and optical frequency signals.^[^ ^45^, ^46^, ^47^, ^48^, ^49^ ^]^ It has broad applications, including frequency stabilization,^[^ ^49^, ^50^ ^]^ optical frequency translation,^[^ ^43^, ^45^, ^47^, ^48^, ^51^, ^52^, ^53^, ^54^ ^]^ Doppler shift analysis of RF signals,^[^ ^52^, ^55^ ^]^ optical gyroscopes,^[^ ^56^, ^57^, ^58^ ^]^ optical atomic clocks,^[^ ^54^, ^59^ ^]^ phase‐locking in atomic interferometers,^[^ ^45^, ^46^, ^51^, ^60^ ^]^ and LIDAR velocimetry.^[^ ^61^, ^62^ ^]^ Apart from simplifying the integration process, serrodyne modulation through EOMs offers several advantages over AOMs, such as a wider tuning range,^[^ ^46^, ^49^ ^]^ faster response time, and lower power consumption.

The use of serrodyne modulation for optical comb frequency shifting has been shown to effectively preserve phase coherence and shift the carrier‐envelope offset frequency^[^ ^63^ ^]^—key characteristics for high‐precision measurement applications. While prior studies have explored the feasibility of integrating optical combs with serrodyne modulation,^[^ ^50^, ^54^, ^64^, ^65^ ^]^ a systematic investigation of its metrological performance in dual electro‐optic frequency comb ranging systems—particularly under practical, non‐ideal modulation regimes—remains lacking. Here, we present a universal framework for AOM‐free dual electro‐optic comb interferometry by incorporating a serrodyne‐modulated EOM into the local oscillator path. Theoretical analysis demonstrates that employing an EOM for frequency shifting enables a 91.84% reduction in RF drive power compared to conventional AOM‐based systems. Experimentally, we validate nanometric ranging precision (Allan deviation is approximately 0.1 nm at 1 ms) across a 19.2 MHz tuning range, successfully resolving high‐frequency vibrations (up to 100 kHz) of dynamic targets. Beyond controlled environments, we demonstrate real‐world applicability by reconstructing 3D profiles of low‐reflectivity targets and monitoring the vibration of water surfaces. By integrating ultralow‐power operation, high‐speed tracking, and compatibility with integrated photonics, this work redefines the scalability of dual electro‐optic comb ranging systems for portable metrology and industrial sensing.

## 2 Results

### 2.1 Measurement Concept

**Figure 1:**
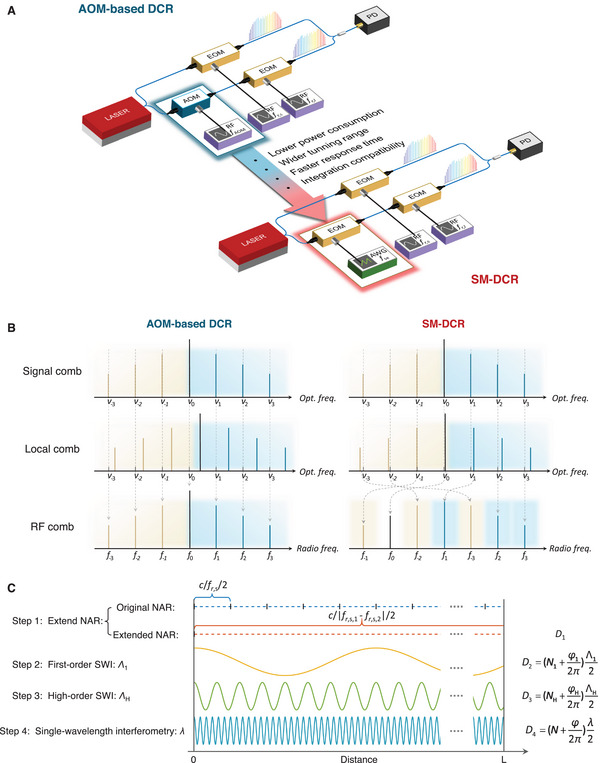
Measurement concept of SM‐DCR system. A) Configurations of AOM‐based (top) and serrodyne‐modulated EOM‐based (bottom) dual comb ranging (SM‐DCR) systems. The SM‐DCR eliminates the need for an AOM by applying sawtooth‐driven serrodyne modulation, enabling lower power consumption, a wider tuning range, a faster response time and enhanced integration compatibility. B) Frequency‐domain representation of optical combs and corresponding RF combs. In the AOM‐based system, symmetric RF spectra are produced around the AOM frequency *f*_AOM_. In contrast, the SM‐DCR system under some parameter configuration exhibits asymmetric RF beat note distributions, as the serrodyne shift *f*_se_ is comparable to or smaller than the repetition rate difference Δ*f*_r_. C) Four‐step hierarchical distance measurement process. Step 1: Extend the non‐ambiguity range (NAR) by switching between two slightly different repetition rates to obtain a coarse absolute distance *D*_1_. Step 2: Perform first‐order synthetic‐wavelength interferometry (SWI) using long synthetic wavelength Λ_1_ to get *D*_2_. Step 3: Apply high‐order SWI with shorter Λ_H_ for refined distance *D*_3_. Step 4: Execute single‐wavelength interferometry using optical phase to achieve high‐precision measurement *D*_4_. In each step, the integer *N* used in *D*_*n*_ is unambiguously determined from the estimate obtained in the previous level *D*_*n* − 1_.

In this work, we refer to dual electro‐optic comb ranging systems as DCR for brevity, as all systems discussed are based on electro‐optic combs and the term is therefore unambiguous. In DCR systems, an AOM is typically placed before the local comb generator. This configuration shifts the central frequency of the seed laser from ν_0_ to ν_0_ + *f*_AOM_, where *f*_AOM_ is the frequency offset introduced by the AOM, as shown in **Figure** **1**A. In the frequency domain (Figure 1B, left panel), the signal comb and local comb are expressed as ν_*n*_ = ν_0_ + *nf*_r, s_ and ν_0_ + *f*_AOM_ + *nf*_r, l_, respectively, where *n* = 0, ±1, ±2, … denotes the mode number relative to the comb center, and *f*_r, s_ and *f*_r, l_ are the repetition rates of the signal and local combs. The difference in repetition rates is defined as Δ*f*_r_ = *f*_r, s_ − *f*_r, l_. Typically, the condition *f*_AOM_ ≫ Δ*f*_r_ is ensured to effectively prevent frequency aliasing, as the beat frequencies in the interference signal are given by *f*_*n*_ = *f*_AOM_ + *n*Δ*f*_r_, where *f*_0_ = *f*_AOM_, thus preserving a one‐to‐one correspondence between optical comb lines and RF comb lines. This mapping ensures that the order of the comb teeth in the optical domain is mirrored in the RF domain, from negative to positive indices.

In the proposed serrodyne‐modulated DCR (SM‐DCR) system, the AOM is replaced by an EOM driven by a sawtooth wave, as illustrated in Figure 1A (bottom panel). This technique, known as serrodyne modulation, applies a continuous linear phase ramp to shift the frequency of the local comb. The sawtooth signal has a period *T*_se_ and repetition frequency *f*_se_ = 1/*T*_se_. An ideal frequency shift is realized when the slope of the phase ramp is 2*m*π*f*_se_, where m∈Z, resulting in:

(1) $$e^{i2\pi f_{0}t}\cdot e^{i2m\pi f_{\text{se}}t}=e^{i2\pi (f_{0}+mf_{\text{se}})t}$$

Thus, the carrier frequency *f*_0_ is shifted by *mf*_se_.

Functionally, serrodyne modulation plays a similar role to AOM‐based shifting in eliminating RF beat note degeneracy. However, due to the ultra‐wide tuning bandwidth of EOMs, the frequency shift *f*_se_ can be flexibly adjusted across a broad range—from a few kilohertz to tens of gigahertz. Compared to AOMs, EOMs also provide superior performance in terms of lower power consumption, faster response time, and enhanced photonic integration compatibility.

If the applied shift satisfies *f*_se_ ≫ Δ*f*_r_, the RF beat notes remain in correct order, and the mapping between optical modes and RF modes remains intact (Figure 1B, left). However, when *f*_se_ ∼ Δ*f*_r_ or smaller, the beat notes may appear scrambled in the RF spectrum (Figure 1B, right), and the correspondence between beat frequencies and optical comb lines must be reestablished during signal processing to ensure correct phase recovery.

For a non‐ideal sawtooth waveform *s*(*t*) with a peak‐to‐peak modulation depth of 2*E*, the phase modulation can be described by its Fourier sine expansion:

(2) $$s\left(t\right)=\frac{2E}{\pi }\sum_{n=1}^{\infty }\frac{{\left(-1\right)}^{n-1}}{n}\sin\left(2\pi nf_{\text{se}}t\right)$$

where *f*_se_ is the modulation frequency. The corresponding complex phase modulation becomes:

(3) $$e^{is(t)}=\exp\left(i\frac{2E}{\pi }\sum_{n=1}^{\infty }\frac{{\left(-1\right)}^{n-1}}{n}\sin\left(2\pi nf_{\text{se}}t\right)\right)$$

Applying the Jacobi–Anger identity,

(4) $$e^{iz\sin(\theta )}=\sum_{m=-\infty }^{\infty }J_{m}\left(z\right)e^{im\theta }$$

here, *J*_*m*_(*z*) denotes the Bessel function of the first kind and order *m*.

Using this identity, the full exponential modulation is expanded into a product of harmonic components:

(5) $$e^{is(t)}=\prod_{n=1}^{\infty }\sum_{m_{n}=-\infty }^{\infty }J_{m_{n}}\left(\frac{2E}{\pi n}\right)e^{i2\pi m_{n}nf_{\text{se}}t}$$

Reorganizing terms yields a spectral decomposition:

(6) $$e^{is(t)}=\sum_{n}C_{n}e^{i2\pi nf_{\text{se}}t}$$

where each coefficient *C*_*n*_ results from products of Bessel functions corresponding to different harmonic contributions:

(7) $$C_{n}=\sum_{\left\{m_{k}\right\}}\prod_{k=1}^{\infty }J_{m_{k}}\left(\frac{2E}{\pi k}\right),\;\text{with}\;\sum_{k=1}^{\infty }km_{k}=n$$

here, *n* denotes the overall harmonic order of the resulting sideband, *k* indexes the harmonic orders of the constituent Fourier components of the sawtooth waveform, and *m*_*k*_ represents the Bessel function order associated with the *k*‐th harmonic component. The constraint $\sum_{k=1}^{\infty }km_{k}=n$ ensures that all combinations of {*m*_*k*_} contributing to *C*_*n*_ yield sidebands at the correct total frequency offset *nf*_se_. This condition arises from the Jacobi–Anger expansion of the multi‐harmonic phase modulation, where each sideband at order *nf*_se_ results from the coherent sum of harmonic contributions *kf*_se_ weighted by Bessel orders *m*_*k*_. This formulation reveals the generation of multiple sidebands centered at harmonics of *f*_se_, with amplitudes governed by weighted combinations of Bessel functions. In the ideal case, the energy is concentrated in a single dominant frequency component; however, residual waveform distortion leads to power leakage into undesired sidebands, reducing spectral purity.

As illustrated in Figure 1C, a four‐step hierarchical strategy is adopted to achieve high‐precision, unambiguous distance measurements in this research.

1. Step 1: Coarse ranging with extended NAR. The intrinsic non‐ambiguity range (NAR) of DCR systems is given by NAR_0_ = *c*/*f*_r, s_/2, which is 9.347 mm in this work. To increase the NAR, we alternate between two signal comb repetition rates *f*_r, s, 1_ and *f*_r, s, 2_, enabling coarse distance estimation over:^[^ ^66^, ^67^, ^68^ ^]^
(8) $${\mathrm{NAR}}_{\text{ext}}=\frac{c}{2|f_{\text{r,s,1}}-f_{\text{r,s,2}}|}$$
  The resulting coarse distance *D*_1_ resolves ambiguity over this extended range.
2. Step 2 & 3: Synthetic‐wavelength interferometry. The interferometric signal is described as:
(9) $$E_{\text{M/R}}=\sum_{i}E_{\text{M/R},i}\cos\left[2\pi \left(f_{\text{se}}+i\Delta f_{r}\right)t+\phi_{\text{M/R},i}\right]$$
  where $E_{\text{M/R}}$ denotes the interference signal from the measurement (M) or reference (R) path, the amplitudes of the beat notes are represented by *E*_M, *i*_ and *E*_R, *i*_ and *i* is the comb mode index. The phase of each component is:
(10) $$\phi_{\text{M/R},i}=\frac{4\pi L_{\text{M/R}}}{\lambda_{i}}$$
  here, λ_*i*_ = *c*/[*n*_*i*_(ν_0_ + *if*_r, s_)] denotes the effective wavelength corresponding to the *i*‐th comb tooth in air, where *n*_*i*_ is the refractive index at that frequency, typically calculated using the Ciddor equation.^[^ ^69^, ^70^ ^]^ *L*_M/R_ is the optical path length of the measurement (M) or reference (R) arm, including both free‐space and fiber segments. The distance is calculated from the phase difference:
(11) $$\phi_{i}=\phi_{M,i}-\phi_{R,i},\;D_{2}=\frac{\lambda_{i}}{2}\cdot \frac{\phi_{i}}{2\pi }$$
  Since multiple λ_*i*_ exist, two beat frequencies can be selected to construct a synthetic wavelength:
(12) $$\Lambda_{pq}=\frac{\lambda_{p}\lambda_{q}}{|\lambda_{p}-\lambda_{q}|}=\frac{c}{\left(q-p\right)n_{g}f_{\text{r,s}}}$$
  where *n*_g_ is the group refractive index of air, estimated from the refractive indices *n*_*p*_ and *n*_*q*_ at wavelengths λ_*p*_ and λ_*q*_ using
(13) $$n_{g}=n_{q}-\lambda_{q}\cdot \frac{n_{q}-n_{p}}{\lambda_{q}-\lambda_{p}}$$
  When |*q* − *p*| = 1, the resulting synthetic wavelength Λ_1_ corresponds to first‐order SWI. The measured distance *D*_2_, refined from the coarse estimate *D*_1_, is then given by:
(14) $$D_{2}=\left(N_{1}+\frac{\varphi_{1}}{2\pi }\right)\frac{\Lambda_{1}}{2},\;N_{1}=\left\lfloor \frac{D_{1}}{\Lambda_{1}/2}\right\rfloor$$
  where φ_1_ = ϕ_*p*_ − ϕ_*q*_, ⌊ · ⌋ is a floor operation. For higher precision, mode pairs with larger separation |*q* − *p*| > 1 are used to construct a shorter synthetic wavelength Λ_H_. This yields the high‐order SWI result:
(15) $$D_{3}=\left(N_{H}+\frac{\varphi_{H}}{2\pi }\right)\frac{\Lambda_{H}}{2},\;N_{H}=\left\lfloor \frac{D_{2}}{\Lambda_{H}/2}\right\rfloor$$
  here, $\varphi_{H}=\phi_{p^{\prime }}-\phi_{q^{\prime }}$ is the phase difference between a higher‐order beat pair (*p*′, *q*′). This step resolves finer phase detail while relying on the ambiguity range resolved by *D*_2_.
3. Step 4: Single‐wavelength interferometry. Finally, individual comb lines are used for phase‐resolved distance extraction:
(16) $$D_{4}=\left(N+\frac{\varphi }{2\pi }\right)\frac{\lambda }{2},\;N=\left\lfloor \frac{D_{3}}{\lambda /2}\right\rfloor$$
  The integer *N* is determined from *D*_3_. Since the single‐wavelength NAR is about λ/2 = 532~nm, this step defines the final resolution. Multiple measurements from different comb lines are averaged to enhance precision and robustness.

### 2.2 Experimental Setup

**Figure 2:**
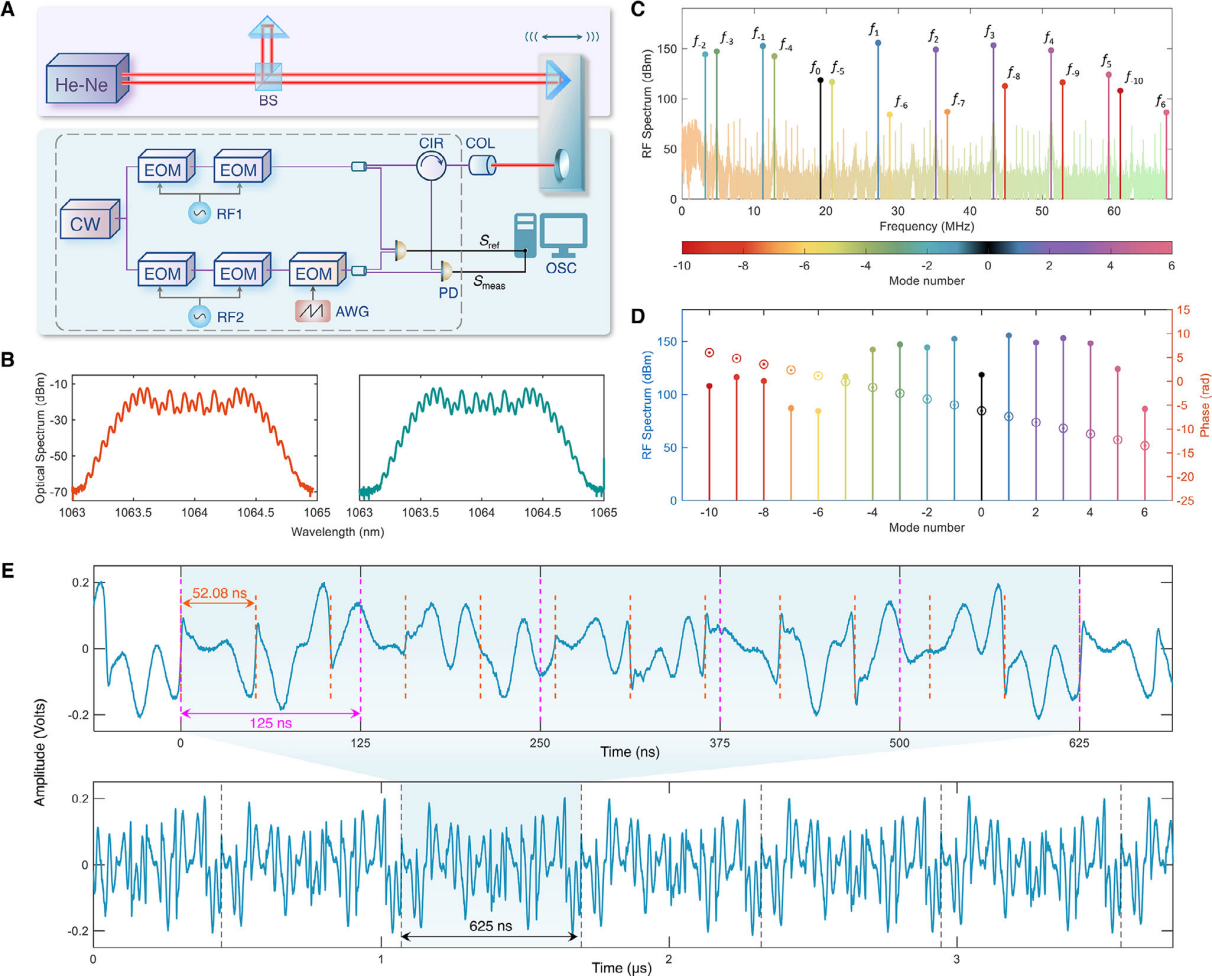
Experimental setup and signals of the dual comb ranging system. A) Experimental setup. The blue section denotes the implemented DCR system, whose results are compared to the He‐Ne interferometer in the purple section. BS: beam splitter; CW: continuous‐wave laser; EOM: electro‐optic modulator; RF: radio frequency source; AWG: arbitrary waveform generator; CIR: circulator; COL: collimator; PD: photodetector; OSC: oscilloscope. B) Optical spectra of the signal comb (left) and local comb (right). C) Interferometric RF spectrum, showing beating components at *n*Δ*f*_r_ + *f*_se_, with colors indicating mode index *n*. D) Reordered RF components from (C), with power and phase spectra shown on the left and right vertical axes, respectively. E) Time‐domain interferogram under 19.2 MHz serrodyne modulation. The upper panel shows zoomed‐in features, revealing nested periodicities: 52.08 ns (serrodyne modulation, orange dashed lines) and 125 ns (beat period, magenta dashed lines). Their least common multiple, 625 ns, is marked in the lower panel with gray dashed lines, indicating the global repetition period.

The experimental setup is illustrated in **Figure** **2**A. The seed laser (1064 nm) is split into two beams, which are independently modulated by two distinct radio frequency (RF) signals to generate the signal and local electro‐optic combs. The repetition rates of the signal and local oscillator are 16.040 GHz and 16.048 GHz, respectively. An additional frequency shift is applied to the local comb using an EOM, driven by a sawtooth waveform with a power of 81.6 mW. In our experiment, the EOM is placed after comb generation, its function is theoretically equivalent to placing it beforehand. All RF signals are synchronized to a single rubidium atomic clock. The fiber components are placed in an enclosure with temperature control and vibration isolation, which is indicated by the gray dashed box in the figure. Subsequently, each comb is split into two arms to generate the measurement interference signal (*S*_meas_) and the reference interference signal (*S*_ref_). The test target is a mirror mounted on a motorized translation stage, which also holds a corner‐cube reflector. The displacement of this reflector is recorded by a commercial He‐Ne laser interferometer, serving as a reference for validating the DCR system's distance measurements.

The optical spectra of the signal and local combs are shown in Figure 2B. By leveraging EOM‐based phase modulation, 23 signal comb lines were produced with a spectral range of approximately 353 GHz, alongside 23 local comb lines covering about 351 GHz. The local comb is frequency‐shifted using serrodyne modulation with sawtooth waveforms at 10 kHz and 19.2 MHz. Due to waveform imperfections and slight deviation of the modulation depth from integer multiples of 2π, unwanted sidebands appear in addition to the desired shifted comb lines at ν_0_ + *nf*_r, l_ + *f*_se_. Nevertheless, subsequent experimental results demonstrate that these weak sidebands do not compromise the phase coherence of the comb teeth in the interference signals.

The spectrum of the dual comb interference signal is shown in Figure 2C, highlighting beat notes located at *n*Δ*f*_r_ + *f*_se_, where Δ*f*_r_ = 8~MHz and *f*_se_ = 19.2~MHz. Different colors represent mode indices *n*, with *n* = 0 marked in black. The central beat note (*n* = 0) is typically excluded from phase measurements as its phase information is degraded, as also reported in previous studies.^[^ ^67^, ^71^, ^72^ ^]^ These beat notes are reordered according to their mode indices and plotted in ascending order in Figure 2D. The observed linear phase progression confirms excellent phase coherence across the comb lines, even after frequency shifting via serrodyne modulation, thereby supporting high‐precision distance measurements.

To further analyze the interferometric signal in the time domain, Figure 2E shows the temporal waveform under 19.2 MHz serrodyne modulation. In the upper panel, phase discontinuities (marked by orange dashed lines) occur clearly at intervals of approximately 52.08 ns (corresponding to the serrodyne modulation frequency *f*_se_ = 19.2~MHz), directly indicating the presence of serrodyne phase modulation. Meanwhile, the fundamental interference period of 125 ns, determined by Δ*f*_r_ = 8~MHz, is marked by magenta dashed lines. Consequently, these two periods jointly lead to a global repetition period of 625 ns, which contains exactly 12 cycles of 52.08 ns and 5 cycles of 125 ns, thus representing their least common period. This global period is clearly observable in the lower panel and marked by gray dashed lines.

### 2.3 Absolute Distance Measurement with a Static High‐Reflectivity Target

To evaluate the absolute ranging performance of the proposed DCR system under ideal conditions, we conducted static distance measurements using a high‐reflectivity mirror mounted on a motorized translation stage. A commercial He‐Ne laser interferometer was employed simultaneously to measure a second mirror mounted on the same stage, enabling direct comparison between the interferometer and the DCR results. The translation stage moved in increments of 1 m, and at each of the ten positions, we acquired 50 consecutive distance measurements using an oscilloscope. The mean and standard deviation of measurement discrepancies between the DCR system and the He‐Ne interferometer at each position were computed and illustrated with one‐standard‐deviation error bars.

**Figure 3:**
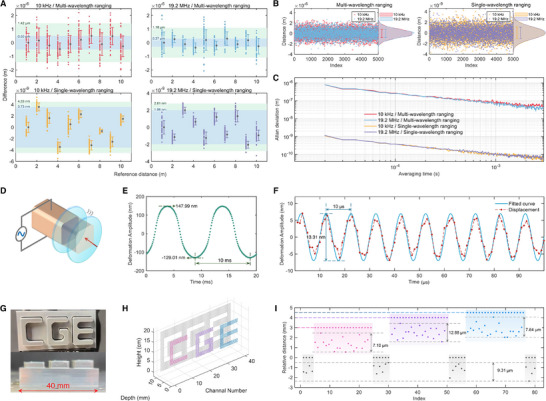
Results of absolute distance measurements, PZT deformation analysis and depth imaging. A) Absolute distance measurement results of the translation stage, including measurements driven by low‐frequency (10 kHz) and high‐frequency (19.2 MHz) sawtooth waveforms, as well as multi‐wavelength and single‐wavelength ranging. The measurement results are presented as scatter plots with error bars on the right. The blue and green shaded regions indicate the accuracy and uncertainty ranges, respectively. B) Long‐term absolute distance measurement results of the translation stage. C) Allan deviation analysis derived from the data in panel B. D) Schematic illustration of PZT deformation, where the longitudinal length of the PZT varies with the applied transverse voltage. E) Measured PZT deformation amplitude at an applied voltage of 100 Hz, with a measurement rate of 10 kHz. F) Measured PZT deformation amplitude at an applied voltage of 100 kHz, with a measurement rate of 1 MHz. G) Target object for depth measurement: the CGE workpiece and its side view showing the depth distribution. H) 3D imaging results of the sample, where different depths are color‐coded. I) Detailed depth distribution of the sample.

The multi‐wavelength ranging results obtained with serrodyne modulation frequencies of 10 kHz and 19.2 MHz are presented in the first row of **Figure** **3**A. The blue shaded areas indicate the range of mean values, while the green shaded areas represent measurement uncertainties. The mean deviations from the He‐Ne interferometer are 0.50 μm (10 kHz) and 0.37 μm (19.2 MHz), which may result from spatial mismatch between the DCR system and the interferometer, target instability, and the limitations of data processing. These results reveal uncertainties of 1.42 μm (10 kHz) and 1.16 μm (19.2 MHz), mainly influenced by the repetition rate stability, the accuracy of refractive index compensation, and the employed signal processing algorithms. To further assess system stability, continuous measurements were performed on a stationary mirror, with the Allan deviation of these distance measurements calculated. Figure 3B (left) shows scatter plots, probability distribution histograms, and corresponding one‐standard‐deviation error bars based on 5000 data points. Red and blue markers correspond to modulation frequencies of 10 kHz and 19.2 MHz, respectively, with standard deviations of 0.82 μm (10 kHz) and 0.79 μm (19.2 MHz). Figure 3C illustrates the Allan deviation curves, both showing approximately 0.80 μm at 20 μs, decreasing to 0.27 μm at 100 μs, and further reducing below 0.10 μm (100 nm) at 1 ms.

Given the laser wavelength of approximately 1064 nm used in this system, the single‐wavelength non‐ambiguity range is about 532 nm. Once the multi‐wavelength ranging uncertainty falls below 266 nm, it becomes feasible to bridge directly to single‐wavelength interferometry. We thus conducted high‐resolution single‐wavelength ranging under both modulation conditions (second row of Figure 3A). The observed mean deviations from the interferometer reference are 3.73 nm (10 kHz) and 1.96 nm (19.2 MHz), with corresponding uncertainties of 4.33 and 2.81 nm, respectively, indicating nanometer‐level precision in both cases. Continuous measurements were similarly performed to evaluate long‐term stability, with Allan deviation analysis conducted. Figure 3B (right) illustrates scatter plots, histograms, and error bars based on 5000 data points, marked in yellow (10 kHz) and purple (19.2 MHz), with standard deviations of 1.11 nm and 1.06 nm, respectively. The Allan deviation curves in Figure 3C demonstrate stability approaching 1.09 nm at 20 μs, decreasing to 0.38 nm at 100 μs, and ultimately around 0.10 nm at 1 ms.

Besides achieving high precision, the EOM‐based frequency shifting employed in our setup provides a broad tunability range—from kHz to tens of MHz—with consistent measurement performance. Compared to conventional AOMs requiring approximately 1 W drive power, our serrodyne‐modulated EOM setup consumed only 81.6 mW (7 V peak‐to‐peak into 50 Ω), thereby reducing power consumption by 91.84%.

### 2.4 Vibration Monitoring of a PZT with a High‐Reflectivity Surface

We next evaluated the system's capability to track dynamic displacement by measuring the deformation of a piezoelectric transducer (PZT) under applied voltage. A mirror affixed to the surface of the PZT served as a reflective target. Applying a transverse voltage to the PZT induced longitudinal deformation, causing microscopic displacement of the mirror, as illustrated in Figure 3D. Two test cases were evaluated: at a driving frequency of 100 Hz (Figure 3E), the measured displacement exhibited a sinusoidal waveform with amplitude varying between ‐129.01 nm and 147.99 nm, and frequency of 1/1~ms = 100 Hz; at 100 kHz (Figure 3F), measurements (red scatter points) were fitted with a sinusoidal function (solid blue line), revealing a displacement with peak‐to‐peak value of 13.31 nm and frequency of 1/10μs=100kHz. The system's high‐speed acquisition capability enabled real‐time tracking of nanometer‐scale vibrations, demonstrating its suitability for monitoring high‐speed dynamic targets.

### 2.5 Depth Imaging of a Low‐Reflectivity Object Under Static Conditions

To assess active measurement capability for general targets, we performed depth imaging on a low‐reflectivity object featuring three protruding letters (C, G, and E) on a flat base. A photograph of the object is shown in Figure 3G. The object was mounted on a 2D translation stage for two‐dimensional scanning. The DCR system reconstructed the full 3D surface by recording absolute distance at each point.

The 3D imaging result is shown in Figure 3H. Figure 3I shows a cross‐section taken at a constant height of 13 cm in panel H. This panel illustrates the relative elevations of the three letters with respect to the base: “C” is raised by 3 mm, “G” by 4 mm, and “E” by 4.5 mm. To quantify measurement stability, we magnified the scatter distributions and annotated the full vertical spread (maximum–minimum) for each region: base (9.31 μm), C (7.10 μm), G (12.88 μm), and E (7.84 μm). The results confirm robust depth reconstruction even for low‐reflectivity surfaces, highlighting the system's potential for use in scenarios such as industrial part inspection, surface damage analysis, and 3D scanning of low‐contrast objects.

### 2.6 3D Profiling of a Rotating Fan with Low Reflectivity

**Figure 4:**
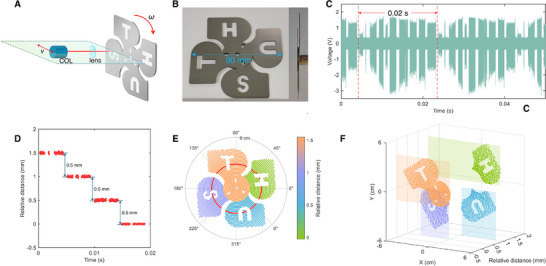
3D reconstruction of a rotating fan. A) Schematic diagram of the experiment for monitoring fan rotation. The collimator and convex lens are co‐mounted on a translation stage and move radially across the fan, parallel to the horizontal plane, with scanning speed *v*. The fan rotates at an angular velocity ω. B) Photographs of the fan: front view (left) showing the blade pattern and side view (right) indicating the axial alignment. C) Periodic interferometric signal with a period of 0.02 s. D) Distance computation results for one signal period in panel C, referenced to the minimum distance within the period (i.e., the fan center distance). E) Polar coordinate representation of the distance data in panel D, with different colors indicating varying distances. F) 3D Cartesian representation of the rotating fan, reconstructed from panel E by incorporating depth information.

To simulate industrial scenarios such as rotating machinery inspection or propeller blade defect detection, we measured both the rotational state and 3D surface profile of a rotating fan using the DCR system. In this experiment, a collimator and convex lens were co‐mounted on a motorized translation stage and scanned radially across the fan plane at a constant velocity *v*, while the fan rotated at an angular speed ω, as illustrated in **Figure** **4**A. This configuration enables the system to capture depth information of rotating targets along a spiral trajectory. Figure 4B shows photographs of the fan, including a front view (blade pattern) and side view (axial alignment). The front view reveals the overall dimensions of the fan to be 90 mm by 90 mm, while the side view highlights a height variation among the fan blades, which was custom‐designed to have a step height of 0.5 mm.

The interferometric signal recorded over a 0.02 s window is presented in Figure 4C, exhibiting periodic features corresponding to a rotation frequency of 50 Hz. Distance data extracted from the red‐shaded interval are plotted in Figure 4D, clearly revealing the designed 0.5 mm height difference between adjacent fan blades, demonstrating the system's capability to resolve sub‐millimeter structural features. Missing data points correspond to hollow regions between blades.

By mapping the time series data into polar coordinates with *r* = *vt* and θ = 2π*ft*, where *v* = 0.08 m/s is the linear scanning speed of the optical system, *f* = 50 Hz is the rotational frequency of the fan, and *t* denotes time, we interpret *r* and θ as the radial and angular coordinates, respectively, in a fan‐centric polar coordinate frame. This transformation enables reconstruction of the spiral measurement trajectory, as shown in Figure 4E, where color represents the relative surface height. This representation clearly captures the contour of each blade and their relative positions. The data were then interpolated and converted into Cartesian coordinates to produce a 3D surface map of the rotating fan, shown in Figure 4F. The resulting point cloud reveals both the macroscopic curvature and fine surface details of the blades.

This experiment highlights the system's ability to perform real‐time, non‐contact monitoring of rotating objects with low reflectivity, demonstrating its suitability for applications in manufacturing quality control, dynamic object inspection, and rotating machinery diagnostics.

### 2.7 Nanometric Vibration Sensing on Oscillating Water Surface

**Figure 5:**
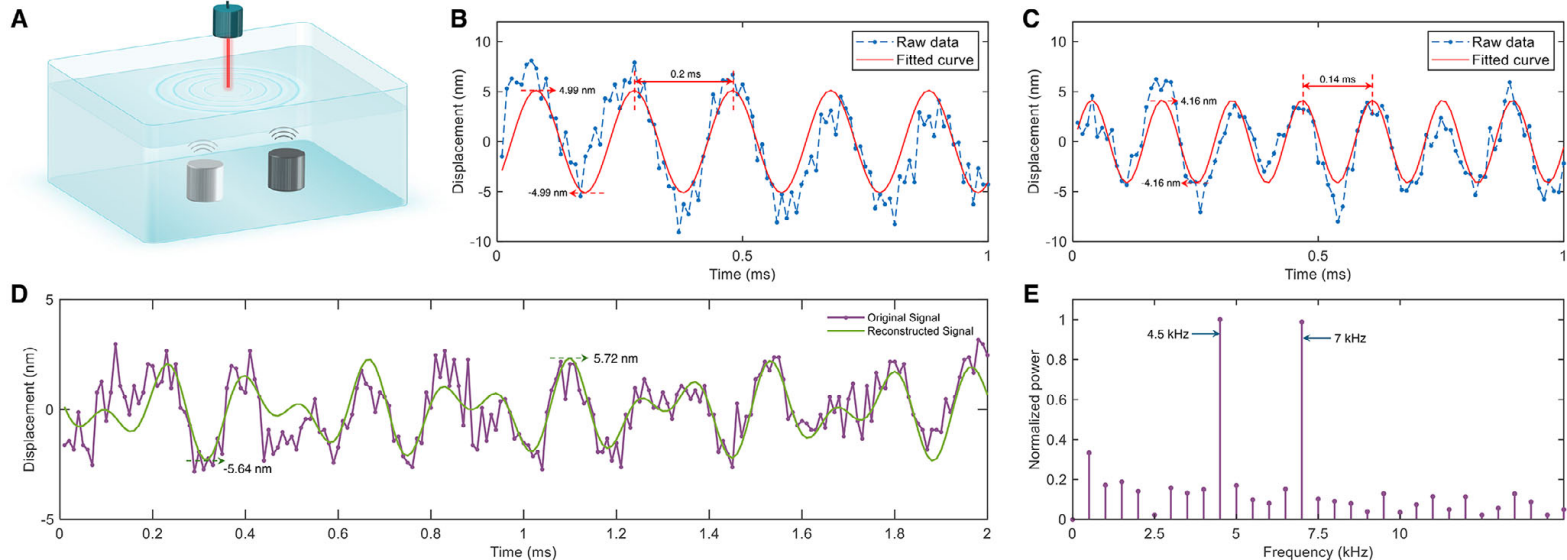
Water surface vibration monitoring. A) Schematic of the water surface vibration measurement setup. Two underwater transmitters induce nanoscale surface displacements detected by a collimated laser beam. B) Time‐domain displacement signal when Transmitter 1 is driven at 5 kHz, showing a clear sinusoidal component. C) Displacement signal for Transmitter 2 at 7 kHz, verifying single‐frequency detection performance. D) Time‐domain signal when both transmitters operate at different frequencies (*f*_drive, 1_ = 4.5 kHz, *f*_drive, 2_ = 7 kHz), demonstrating multi‐frequency resolution. The green trace represents the reconstructed signal using only the two dominant frequency components identified in panel E. E) Frequency spectrum of panel D, with distinct peaks at 4.5 kHz and 7 kHz, confirming effective acoustic separation.

**Figure** **5**A illustrates the schematic of the water surface vibration monitoring experiment. The experiment employs two symmetrically positioned underwater piezoelectric acoustic transmitters, each independently driven at a specific frequency (*f*_drive_) to generate either single‐frequency or multi‐frequency acoustic waves (Transmitter 1: 5 kHz; Transmitter 2: 7 kHz), inducing nanoscale displacement vibration on the water surface. DCR system vertically projects a collimated beam onto the water surface and demodulates the phase variations of the reflected light to capture displacement signals with high precision (measurement speed: 100 kHz).

In the single‐frequency experiment, when only Transmitter 1 is driven (*f*_drive_ = 5 kHz), the time‐domain displacement signal (dashed line in Figure 5B) undergoes sinusoidal fitting, exhibiting a clear 5 kHz vibration component with a fitted amplitude of 4.99 nm. Similarly, when only Transmitter two is activated (*f*_drive_ = 7 kHz), a clear 7 kHz vibration component is observed in the measured signal (solid line in Figure 5B). The fitted sinusoidal curve has a period of approximately 0.14 ms and an amplitude of 4.16 nm, confirming the reliability of single‐frequency vibration monitoring.

To evaluate the system's ability to resolve multiple acoustic sources, both transmitters are simultaneously driven at different frequencies (*f*_drive, 1_ = 4.5 kHz, *f*_drive, 2_ = 7 kHz). The corresponding time‐domain displacement signal is shown in Figure 5D, along with a reconstructed signal (green curve) obtained using only the two dominant frequency components identified in the frequency spectrum (Figure 5E). The reconstructed signal exhibits a maximum amplitude of 5.72 nm and a minimum of –5.64 nm, with a period of 2 ms—corresponding to the least common multiple of the two drive frequencies. This period contains exactly nine cycles of the 4.5 kHz component and 14 cycles of the 7 kHz component, illustrating the system's capability to resolve and faithfully reconstruct multi‐frequency vibration signals. The measured displacement closely follows the trend of the reconstructed signal, highlighting the precision of multi‐frequency vibration monitoring. The frequency spectrum (Figure 5E) reveals distinct peaks at 4.5 kHz and 7 kHz. This result demonstrates that the system effectively distinguishes multiple simultaneous underwater acoustic components through frequency‐domain analysis, and its frequency resolution can be further enhanced by increasing the sampling duration (Δ*f*∝1/*T*_measure_).

This experiment successfully demonstrates the capability of the DCR system for high‐precision, non‐contact vibration monitoring of fluid surfaces, providing a technological basis for acoustic feature extraction and detailed surface vibration analysis.

## 3 Discussion

Based on the DCR system with EOM serrodyne frequency shifting proposed in this study, we achieved high‐precision and wide dynamic‐range absolute distance measurement. Additionally, the system also demonstrated robust performance and adaptability across various experimental scenarios.

Although the tuning range of EOMs can, in principle, extend up to a maximum of tens of gigahertz, this study only demonstrates serrodyne frequency shifting up to 19.2 MHz. This problem stems from the AWG bandwidth rather than the SM‐DCR scheme itself.

Despite the superior measurement performance demonstrated, several challenges and limitations remain in practical applications. For instance, the ideal waveform for serrodyne modulation is difficult to achieve accurately, and non‐ideal waveforms can generate spurious sidebands, potentially increasing system noise and adversely affecting measurement precision. Therefore, future research should focus on optimizing waveform generation circuits and enhancing sideband suppression techniques to further improve the system's signal‐to‐noise ratio and measurement stability.

Furthermore, the experimental setup used in this study imposed strict requirements on temperature and mechanical stability. All optical components, except for the free‐space beam path, were encapsulated within an actively temperature‐controlled and vibration‐damping enclosure, maintaining environmental temperature fluctuations within 1 mK. However, achieving such stringent control remains challenging in industrial or other complex environments. Simplifying system integration and enhancing tolerance to environmental disturbances will be crucial areas for future research to facilitate broader application and implementation.

While this study does not realize a fully integrated prototype, the emphasis on integration readiness is grounded in the fundamental architectural choices of the system. The adoption of EOMs in place of bulky acousto‐optic devices represents a deliberate step toward integration, as EOMs are inherently compatible with photonic integrated circuit platforms. Furthermore, all optical components could be integrated on a photonic chip. The electrical parts can also be integrated onto a single printed circuit board (PCB), while signal acquisition and processing could be implemented using on‐chip ADCs and FPGAs. The comparison of dual‐comb ranging systems based on different frequency offset schemes, as shown in **Table** **1**, demonstrates that our approach combines high ranging precision, fast response, wide frequency‐shifting range, low RF power consumption, and low integration complexity in one system. To further improve the system performance, we consider several potential strategies that can be used, including employing ultra‐narrow‐linewidth lasers, using higher‐stability frequency references, and increasing the measurement speed.

**Table 1:** Comparison of dual‐comb ranging systems based on different frequency offset schemes.

Comb Type	Frequency Shift Scheme	Response Time	Bandwidth	Typical RF Power	Integration Complexity	Precision	Refs.
Dual microcombs	Dual pump lasers	–	–	–	Low	12 nm @ 13 µs	[73]
						32 nm @ 4 µs	[74]
	AOM	∼100 ns	Tens of MHz	1–2 W	High	200 nm @ 500 ms	[75]
						2.8 nm @ 1 s	[76]
Dual EO combs						2 µm @ 9.1 µs	[77]
						3.4 µm @ 14 µs	[78]
						372 nm @ 1.15 ms	[79]
						750 nm @ 167 µs	[67]
						20 µm @ 10 s	[35]
	EOM	∼10 ps	Tens of GHz	<100 mW	Low	<0.1 nm @ 1 ms	This work

## 4 Conclusion

This work introduces a novel dual electro‐optic comb ranging system utilizing a serrodyne‐modulated EOM to achieve frequency shifting, thereby replacing conventional AOMs. This configuration effectively resolves frequency degeneracy issues, reduces power consumption by 91.84% (from approximately 1 W down to 81.6 mW), and maintains exceptional phase coherence. Experimental validations demonstrate that this AOM‐free approach initially achieves multi‐wavelength ranging uncertainties as low as 1.16 μm, subsequently reduced to 2.81 nm through single‐wavelength interferometry, and ultimately achieving sub‐nanometric measurement precision with Allan deviations approximately 0.10 nm at a 1 ms integration time.

The proposed system has exhibited robust performance in various precision metrology applications, successfully tracking dynamic displacements in diverse scenarios, including monitoring PZT vibrations at frequencies up to 100 kHz, reconstructing real‐time 3D images of rotating targets, and detecting nanometric‐level vibrations on fluid surfaces. Notably, the system demonstrates high‐precision distance measurement across a remarkably broad spatial scale—from the initial 10‐meter‐level free‐space ranging, to millimeter‐scale structural profiling, and down to nanometer‐scale vibration sensing on fluid surfaces—highlighting its capability to operate effectively across multiple orders of magnitude. These demonstrations underscore the system's versatility and potential for sophisticated optical sensing applications.

With miniaturization and integration emerging as key trends in optical metrology, electro‐optic frequency combs stand out for their practical features, including simplified generation, flexible modulation schemes, and robust integration capability. Therefore, advancing the integration of dual electro‐optic comb interferometry is of significant interest. The proposed replacement of AOMs with serrodyne‐modulated EOMs aligns with this integration demand while maintaining measurement precision. The AOM‐free configuration provides additional benefits: EOMs offer a wider tuning range, lower RF drive power, faster response time, and reduced complexity in integrated photonic platforms, all of which are crucial for compact ranging systems. Although this work primarily focuses on precision distance measurement, the proposed method is broadly applicable to any dual comb interferometric phase measurement system, offering potential for a wide range of high‐resolution sensing applications.

## Conflict of Interest

The authors declare no conflict of interest.

## Author Contributions

X.‐G. and X.‐Y. contributed equally to this work. X.G. developed the methodology, performed the experiments, prepared the original draft, contributed to manuscript revision, and created the visualizations. X.Y. conducted experiments, developed software tools, carried out formal analysis, and contributed to data visualization. J.Z. contributed to methodology development and software implementation. C.S. led the overall research direction and supervised all stages of the project. H.W. conceived the project, provided supervision and resources, secured funding, coordinated project administration, and revised the manuscript. All authors discussed the results and approved the final version of the manuscript.

## Data Availability

The data that support the findings of this study are available from the corresponding author upon reasonable request.
